# Scale-up of Malaria Rapid Diagnostic Tests and Artemisinin-Based Combination Therapy: Challenges and Perspectives in Sub-Saharan Africa

**DOI:** 10.1371/journal.pmed.1001590

**Published:** 2014-01-21

**Authors:** Guido J. H. Bastiaens, Teun Bousema, Toby Leslie

**Affiliations:** 1 Department of Medical Microbiology, Radboud University Medical Center, Nijmegen, The Netherlands; 2 Department of Immunology and Infection, London School of Hygiene & Tropical Medicine, London, United Kingdom; 3 Department of Disease Control, London School of Hygiene & Tropical Medicine, London, United Kingdom

## Abstract

Guido Bastiaens and colleagues describe barriers to achieving scale-up and appropriate use of rapid diagnostic tests and artemisinin-based combination therapy for malaria in sub-Saharan Africa.

*Please see later in the article for the Editors' Summary*

**Summary Points**

- Scaling up and sustaining access to malaria diagnosis and treatment in all public sector, for-profit, and informal health facilities across sub-Saharan Africa is central to current global strategies for malaria control and elimination.
- The use of malaria rapid diagnostic tests (RDTs) aims to eliminate reliance on signs and symptoms to diagnose and treat malaria but evidence shows health workers do not always test the right patients, nor provide treatment based on the results of the test.
- Expanding access to malaria RDTs on the scale needed to achieve universal coverage requires retraining of public, private, and retail sector providers as well as sustained supplies and quality assurance.
- Barriers to rational use of tests and drugs may be overcome through appropriate policy design for the local health service setting, which addresses health worker practice and patient perceptions.
- Innovative methods have been used to increase access to the most effective antimalarial drugs in the last five years, but these efforts will be incomplete and unsustainable without similar efforts to incorporate RDTs into practice.

## Introduction

An estimated 627,000 malaria deaths occurred in 2012, mostly in African children and many of them preventable with prompt diagnosis and treatment [1]. Access to diagnosis remains poor—in half of endemic African countries, over 80% of malaria treatments are applied without diagnostic testing [2]. Improving diagnosis and treatment of malaria will improve treatment outcomes, rationalize health care costs by reducing drug consumption [3], minimize drug pressure that can contribute to resistance [4],[5], and assist in monitoring disease trends [2].

In April 2012, the World Health Organization's (WHO) Global Malaria Programme launched a highly ambitious new initiative: *T3: Test. Treat. Track* [1],[2]. T3 aims to address the widespread problem of poor access to diagnostic testing and antimalarial treatment, and to enhance case-reporting. It sets a target of universal access to diagnostic testing in the public and private health care sector by 2015 [1],[2]. Achieving this goal will centre on the use of malaria rapid diagnostic tests (RDTs).

In this Policy Forum article we examine the operational challenges to implementing the T3 strategy of scaling up and maintaining RDT coverage. We identify gaps in planning for at-scale implementation in policy design and implementation, the local health care setting, and the attitudes and demands of patients. While focussed on malaria diagnosis and treatment, the challenges illustrated here are not unique to malaria and may apply to health care provision across resource-poor settings.

## Policy Design and Implementation

By 2012, 41 out of 44 endemic countries in the WHO African Region had adopted the policy of providing malaria diagnostic testing for all age groups before treatment [2]. RDT procurement increased worldwide from 45 million units in 2008 to 205 million in 2012 although supply remains far short of requirements [1],[2]. In theory, the availability of reliable easy-to-use tests should result in a switch from presumptive treatment based on signs and symptoms alone, to parasite-based diagnosis and treatment based on test results. Diagnostic processes and treatment decisions are, however, often irrational and health staff do not always test the right patients, nor provide treatment based on the results [6]–[8].

RDTs will be introduced in health facilities and among community health workers (CHWs) who work at local levels. To translate the change in policy to a change in routine practice where tests are appropriately used by providers, unambiguous messages and guidelines that are adapted to the local context are needed [6],[9],[10]. This targeted information must counter the widespread and long-held guidelines that promoted presumptive treatment of malaria in cases of fever [11]. Appropriate information and training will improve implementation at the community level [12]. Recent evidence shows that CHWs reliably provided Integrated Management of Childhood Illnesses to children after training and incorporation of RDTs into the algorithm [13],[14]. In one study, malaria and pneumonia were appropriately classified in 94%–100% of children, and supply management of medications and RDTs was excellent [13]. Replicating these effects outside the trial setting requires national level training to ensure safety and quality of services.

Mobilising sufficient resources for the training and monitoring required to sustain the new policy is the key to success. A reliable system for RDT delivery needs to include re-training of staff and consistent quality assurance at all levels. The quality of services is likely to wane over time and can be aggravated by high staff turnover, which occurs in many health service settings. Ensuring programme quality and sustainability therefore requires constant rolling interventions and local evidence for the best models of implementation.

## The Local Health Care Setting

In the local health care setting, two problems persist: firstly, parasite-based testing is generally unavailable [1],[2] with treatment decisions based on clinical signs and symptoms that are neither sensitive nor specific [15]; and secondly, if tests are available, health workers often do not apply treatment according to the result of the test [10],[16]–[18]. Both situations result in extensive overuse of antimalarial drugs, especially in low transmission settings [19],[20].

When RDTs are introduced in presumptive treatment settings significant reductions in the overprescription of antimalarials have been seen in almost all studies published (Table S1). However, when they are introduced in settings that have used microscopic examination of blood smears, the advantages of RDTs are harder to define. Substantial numbers of patients may still be treated with an antimalarial drug despite a negative RDT or blood smear result, so the evidence of any clinical advantage of RDTs over microscopy is unclear in some settings (Table S2).

Often, the irrational use of tests and drugs is based on perceived shortcomings of the tests. A common concern amongst health staff is that negative tests do not definitively rule out malaria [21], but trials that withheld antimalarials in febrile children with negative test results have shown no additional malaria risk to patients in moderate-to-high transmission settings. In one trial in Uganda, 13/1,602 (0.8%) blood smear–negative patients who were not given antimalarial drugs developed clinical malaria over 7 days of follow-up and all 13 were detected by the health service and treated [22]. Similar findings were seen in Tanzania (3/603 [0.5%] of RDT-negative patients developed malaria within 7 days) [23]. These studies indicate that withholding antimalarial therapy in febrile children with negative test results is likely to be safe and results in a considerable reduction in antimalarial drug consumption.

Improvements in antimalarial prescription often coincide with increases in prescription of antibiotics amongst test-negative patients. All studies where antimalarial prescription rates were reduced in malaria-negative patients show an increase in antibiotic prescriptions (Tables S1 and S2) [16],[19],[24]–[26]. There is little data on the spectrum of infections in patients presenting with symptoms of suspected malaria but most of these are probably self-limiting [23],[27], and evidence that supports the prevailing practice of widespread antibiotic use in malaria negative patients is lacking.

Identifying patients at risk of progressing to severe disease in which antibiotic treatment and/or referral would have a clinical advantage, while withholding antibiotic treatment in other patients, is a considerable challenge. Affordable rapid diagnostics for bacterial infections or markers of severe infections would support the rational prescription of both antimalarials and antibiotics.

### Patient load and malaria diagnosis

A high patient load in many clinics creates challenges in implementing new policies and motivating staff [28],[29]. In Tanzania, health workers identified high patient load and shortage of staff as key factors that hindered use of RDTs [28]. Most staff felt RDTs placed additional strain on normal operations and believed more staff were needed to conduct the tests [28]. Although these considerations apply to all diagnostic procedures and are not unique to RDTs, understanding the realities of routine practice is required because introducing extra staff into facilities will have an impact on cost.

### Sustained supply of RDTs in public and private sectors

Sustaining the supply of RDTs is a substantial challenge. In rural areas, where access to services is often low but demand for services may be highest [1], drug stock-outs are common [30],[31] and supply is one of the biggest challenges facing the health system. The T3 recommendations imply that a constant supply of both artemisinin-based combination therapies (ACTs) and RDTs is needed. The shelf-life and performance of both diagnostics and drugs depends on their storage conditions; RDTs are degraded by high temperatures and humidity and the entire supply chain must ensure that RDTs remain within manufacturers' recommended limits. WHO testing of a range of commercially available RDTs demonstrated consistent detection of malaria at tropical temperatures [21], but actual field data on storage conditions affecting RDT stability are scarce.

The private for-profit sector plays an important role in delivering services across most of Africa and the majority of suspected malaria episodes are initially treated by private health workers [32],[33]. Data from a limited number of countries suggest neither microscopy nor RDTs have penetrated the private health care sector [1],[34] but more than 50% of patients purchase drugs from unregistered shops and peddlers [32],[33]. This occurs especially amongst lower income groups [35]. Improving diagnostic and treatment practices in the private sector could have a substantial impact on access to diagnosis before treatment but models of implementation have yet to be fully assessed in operational trials [35],[36].

### Affordability and cost-effectiveness of RDT-based diagnosis

To improve access to drugs in sub-Saharan Africa, the *Affordable Medicines Facility - malaria* provided subsidised ACT drugs in a multi-country pilot [37]. This study demonstrated improved access and market share of ACTs in five out of seven pilot countries driven mainly by improvements in the private for-profit sector [38]. In 2012, 331 million courses of ACTs were procured by the public and private sectors in endemic countries, up from 182 million in 2010 [1]. Although the pilot rapidly improved availability, affordability, and market share of quality-assured ACTs at the point of use, no equivalent increase in RDTs has been observed [1],[38]. As diagnosis is seldom available and ACT orders are more than double that of RDTs, overtreatment is likely to be common in retail outlets. ACTs are approximately ten times more costly than previously used monotherapies [19],[31] so the use of RDTs prior to treatment may improve cost-effectiveness. Data from a willingness-to-pay study in private drug shops in Uganda indicated that there was a demand for RDTs in the private sector but this was far below the price of delivery [39]. Subsidised supply of RDTs, similar to the ACTs subsidy, should be assessed to examine the impact on the uptake of RDTs in the private retail sector.

In high and very high transmission areas, presumptive treatment has cost-effectiveness advantages given the imperfect sensitivity of tests under field conditions [3]. RDTs in settings with up to 62% *Plasmodium falciparum* prevalence were cost-effective compared to presumptive treatment, assuming that prescribers adhered fully to test results [31]. When treatment is consistent with the results of a test, cost savings of between 50% and 100% can be achieved compared with presumptive treatment [3]. Conversely, if treatment is inconsistent with the result of the test, cost-effectiveness is reduced, an association that varies with the malaria transmission setting [3],[31]. Other factors that can reduce cost-effectiveness are stock-outs, poor accuracy of RDTs, and poor quality assurance for drugs and diagnostics [31].

In low-endemic settings, RDTs and microscopy remain attractive compared to presumptive treatment even when there is poor adherence to negative test results [3]. RDTs can be more cost-effective than microscopy because they are more accurate under real-life conditions [31] and continuous (re-)training of microscopists is particularly important if fewer malaria positive slides with low parasite levels are encountered in low-endemic settings. Despite these advantages of RDTs over presumptive treatment, adherence to microscopy and RDT test results remains a key factor for cost-effective diagnosis and treatment [3],[40].

### Malaria diagnosis in elimination programmes

Currently available RDTs will not detect all infections with low parasite loads. These submicroscopic infections frequently occur in low-endemic areas [41], are probably not associated with clinical risks [42], but do play a role in onward malaria transmission [43]. Diagnostics with a sensitivity that is higher than currently available RDTs will be needed to identify all malaria infections in elimination efforts [44]. Operational approaches may involve screening by RDT to identify geographic or demographic clusters of infections [45],[46] that can be targeted following molecular diagnosis of infection or by focal mass drug administration [47],[48].

## Attitudes and Demands of Patients

Patients can influence the diagnostic and treatment practices of health workers [7],[8] and patient pressure on providers contributes to overtreatment [7]. There is a persistent perception that all fever episodes in malaria endemic areas are due to malaria [49] and, until recently, a global policy of presumptive treatment for malaria in cases of fever has been in place [2]. These factors have created entrenched demand for malaria treatment without first testing for malaria [29],[50],[51]. Efforts to change demands to promote malaria testing are particularly important in the private and informal sector, where few patients presently receive a diagnostic test. A change in public perceptions brought about by effective communication is needed to widen demand for testing before treatment.

## Conclusions

Meeting the global target of universal coverage with parasite-based diagnosis by 2015 is a huge undertaking requiring sufficient resources. The cost-effectiveness of the intervention will hinge on the accurate use of RDTs in guiding treatment. Probably the biggest challenge in RDT implementation will be to provide adequate and sustained supplies of RDTs and appropriate training to all health workers in endemic areas. With increased access to malaria diagnosis, there will also be increased use of antibiotics, and interventions to guard against even greater overuse are needed to prevent worsening antimicrobial resistance. The *Affordable Medicines Facility - malaria* initiative demonstrated that large increases in access to ACTs were possible. Increasing access to RDTs is equally important. ACTs and RDTs should be seen as a package to improve management of febrile cases, and improving access to both of these in the public and private sectors has the potential to provide valuable returns.

## Supporting Information

Table S1 **Patients treated with antimalarials and antibiotics in studies comparing clinical diagnosis with RDTs.**
(DOC)

Table S2 **Patients treated with antimalarials and antibiotics in studies comparing microscopy with RDTs.**
(DOC)

## Abbreviations

ACT: artemisinin-based combination therapy
CHW: community health worker
RDT: malaria rapid diagnostic test
WHO: World Health Organization
