# Supplementary material for: Scale-up of Malaria Rapid Diagnostic Tests and Artemisinin-Based Combination Therapy: Challenges and Perspectives in Sub-Saharan Africa
Source: PLoS Med. 2014 Jan 21;11(1):e1001590. doi: 10.1371/journal.pmed.1001590 (PMC3897367; doi:10.1371/journal.pmed.1001590)
Supplement: Table S1 — Patients treated with antimalarials and antibiotics in studies comparing clinical diagnosis with RDTs. (DOC) [file pmed.1001590.s001.doc]

**Table S1.** Patients treated with antimalarials and antibiotics in studies comparing clinical diagnosis with RDTs

| **Study** | **Country** | **Study design** | **Treatment with antimalarials** | | | **Treatment with antibiotics** | |
| --- | --- | --- | --- | --- | --- | --- | --- |
|  |  |  | **Clinical diagnosis** | **RDT** | | **Clinical diagnosis** | **RDT** |
|  |  |  | **% (95%CI), n/N** | **% of RDT-positives** | **% of RDT-negatives** |  | **% of RDT-negatives** |
| 1 | Tanzania | Pilot implementation study | 61.3 (57.4-65.3), 365/595 | 98.4 (97.7-99.0), 1329/1351 | 24.7 (22.3-27.2), 289/1168 | NR | NR |
|  |  |  |  |  |  |  |  |
| 2 | Tanzania | Cross-over validation trial | 85.3 (82.9-87.6), 752/882 | 35.9 (33.0-38.9), 361/1005 | | 26.6 (23.7-29.6), 235/882 | 37.0 (34.0-40.0), 372/1005^a^ |
|  |  |  |  |  | |  |  |
| 3 | Burkina Faso | Randomized, multi-centre, open clinical trial | 87.2 (85.3-89.2), 969/1111 | 97.7 (96.4-98.9), 543/556 | 81.0 (77.5-84.4), 400/494 | 54.8 (51.9-57.7), 609/1111 | 56.9 (52.5-61.2), 281/494 |
|  |  |  |  |  |  |  |  |
| 4 | Uganda | Quasi-experimental implementation study | 54.3 (53.9-54.7), 29117/53629 | 32.9 (32.5-33.2), 24591/74784^a^ | | NR | NR |
|  |  |  |  |  | |  |  |
| 5 | Ghana | Randomized controlled open label trial | 92.7 (91.5-94.0), 1598/1723 | 99.6 (99.1-100.0), 703/706 | 49.5 (46.4-52.5), 501/1013 | 22.3 (20.3-24.3), 384/1723 | 35.0 (32.1-38.0), 355/1013 |
|  |  |  |  |  |  |  |  |
| 6 | Malawi | Cross-sectional survey | NR | 98.0 (97.1-98.9), 985/1005 | 57.9 (53.0-62.9), 223/385 | NR | NR |
|  |  |  |  |  |  |  |  |
| 7 | Tanzania | Observational study | NR | 97.1 (96.7-97.6), 5331/5488 | 4.0 (3.4-4.5), 205/5162 | NR | NR |
|  |  |  |  |  |  |  |  |
| 8 | Zambia | Cluster randomized controlled trial | 99.1 (98.7-99.5),  2066/2084 | 27.5 (24.7-30.3), 265/963^b^ | | NR | NR |
|  |  |  |  |  | |  |  |
| 9 | Zambia | Prospective pilot study | NR | 100.0, 2318/2318 | 0.6, 13/NR and 0.0, NR^c^ | NR | NR |
|  |  |  |  |  |  |  |  |
| 10 | Tanzania | Randomized cross-over trial | 96.5 (95.6-97.5), 1422/1473 | 99.7 (99.3-100.0), 731/733 | 6.1 (4.3-7.8), 44/724 | NR | NR |
|  |  |  |  |  |  |  |  |
| 11 | Tanzania | Longitudinal study | 98.6 (98.3-98.9), 4448/4511 | 31.8 (31.1-32.6), 4440/13942 | | NR | NR |
|  |  |  |  |  | |  |  |
| 12 | Nigeria | Cross-sectional study | NR | 100.0, 92/92 | 74.1, (65.8-82.3) 80/108 | NR | NR |
|  |  |  |  |  |  |  |  |
| 13 | Burkina Faso | Open cluster randomized trial | 99.8 (99.5-100.0),  575/576 | 99.2 (98.4-100.0), 388/391 | 3.7 (0.5-6.9), 5/134 | NR^d^ | 59.0 (50.6-67.3), 79/134 |
|  |  |  |  |  |  |  |  |
| 13 | Ghana | Open cluster randomized trial | 95.3 (93.5-97.0),  563/591 | 100.0, 492/492 | 3.3 (0.0-6.9), 3/92 | 64.3 (60.4-68.2), 380/591 | 69.6 (60.2-79.0), 64/92 |
|  |  |  |  |  |  |  |  |
| 13 | Uganda | Open cluster randomized trial | 100.0,  965/965 | 99.9 (99.7-100.0), 856/857 | 7.6 (2.8-12.4), 9/118 | NR^d^ | 59.3 (50.5-68.2), 70/118 |
|  |  |  |  |  |  |  |  |
| 14 | Uganda | Cross-sectional survey | 60.1 (59.3-60.9), 8432/14024 | 32.7 (32.0-33.5), 4892/14940 | | NR | NR |

RDT, malaria rapid diagnostic test; NR, not reported

^a^ 99% of RDT-positives received antimalarials, 30% of febrile RDT-negatives received antimalarials

^b^ 3 out of 704 RDT-negatives received antimalarials

^c^ Data of two districts

^d^ Antibiotics not available as treatment

**Reference list**

1. Williams HA, Causer L, Metta E, Malila A, O'Reilly T, et al. (2008) Dispensary level pilot implementation of rapid diagnostic tests: an evaluation of RDT acceptance and usage by providers and patients--Tanzania, 2005. Malar J 7: 239.

2. Msellem MI, Martensson A, Rotllant G, Bhattarai A, Stromberg J, et al. (2009) Influence of rapid malaria diagnostic tests on treatment and health outcome in fever patients, Zanzibar: a crossover validation study. PLoS Med 6: e1000070.

3. Bisoffi Z, Sirima BS, Angheben A, Lodesani C, Gobbi F, et al. (2009) Rapid malaria diagnostic tests vs. clinical management of malaria in rural Burkina Faso: safety and effect on clinical decisions. A randomized trial. Trop Med Int Health 14: 491-498.

4. Kyabayinze DJ, Asiimwe C, Nakanjako D, Nabakooza J, Counihan H, et al. (2010) Use of RDTs to improve malaria diagnosis and fever case management at primary health care facilities in Uganda. Malar J 9: 200.

5. Ansah EK, Narh-Bana S, Epokor M, Akanpigbiam S, Quartey AA, et al. (2010) Rapid testing for malaria in settings where microscopy is available and peripheral clinics where only presumptive treatment is available: a randomised controlled trial in Ghana. BMJ 340: c930.

6. Chinkhumba J, Skarbinski J, Chilima B, Campbell C, Ewing V, et al. (2010) Comparative field performance and adherence to test results of four malaria rapid diagnostic tests among febrile patients more than five years of age in Blantyre, Malawi. Malar J 9: 209.

7. Masanja MI, McMorrow M, Kahigwa E, Kachur SP, McElroy PD (2010) Health workers' use of malaria rapid diagnostic tests (RDTs) to guide clinical decision making in rural dispensaries, Tanzania. Am J Trop Med Hyg 83: 1238-1241.

8. Yeboah-Antwi K, Pilingana P, Macleod WB, Semrau K, Siazeele K, et al. (2010) Community case management of fever due to malaria and pneumonia in children under five in Zambia: a cluster randomized controlled trial. PLoS Med 7: e1000340.

9. Chanda P, Hamainza B, Moonga HB, Chalwe V, Pagnoni F (2011) Community case management of malaria using ACT and RDT in two districts in Zambia: achieving high adherence to test results using community health workers. Malar J 10: 158.

10. Mubi M, Janson A, Warsame M, Martensson A, Kallander K, et al. (2011) Malaria rapid testing by community health workers is effective and safe for targeting malaria treatment: randomised cross-over trial in Tanzania. PLoS One 6: e19753.

11. Ishengoma DS, Francis F, Mmbando BP, Lusingu JP, Magistrado P, et al. (2011) Accuracy of malaria rapid diagnostic tests in community studies and their impact on treatment of malaria in an area with declining malaria burden in north-eastern Tanzania. Malar J 10: 176.

12. Uzochukwu BS, Onwujekwe E, Ezuma NN, Ezeoke OP, Ajuba MO, et al. (2011) Improving rational treatment of malaria: perceptions and influence of RDTs on prescribing behaviour of health workers in southeast Nigeria. PLoS One 6: e14627.

13. Mukanga D, Tiono AB, Anyorigiya T, Kallander K, Konate AT, et al. (2012) Integrated community case management of fever in children under five using rapid diagnostic tests and respiratory rate counting: a multi-country cluster randomized trial. Am J Trop Med Hyg 87: 21-29.

14. Kyabayinze DJ, Asiimwe C, Nakanjako D, Nabakooza J, Bajabaite M, et al. (2012) Programme level implementation of malaria rapid diagnostic tests (RDTs) use: outcomes and cost of training health workers at lower level health care facilities in Uganda. BMC Public Health 12: 291.

**Search strategy**

On 24 April 2013 we searched the PubMed electronic database to locate relevant RDT-implementation studies from sub-Saharan Africa, focusing on intervention and observational studies of non-RDT versus RDT and pre- versus post-RDT implementation. We used the following combination of search terms:

Search #1: (“case management”[Text Word]) AND (“malaria”[Text Word]) AND (“rapid diagnostic test”[Text Word])

Search #2: (“treatment practices”[Text Word]) AND (“malaria”[Text Word]) AND (“rapid diagnostic test”[Text Word])

Search #3: (“implementation”[Text Word]) AND (“malaria”[Text Word]) AND (“rapid diagnostic test”[Text Word])

Search #4: (“treatment outcome”[Text Word]) AND (“malaria”[Text Word]) AND (“rapid diagnostic test”[Text Word])

Search #5: (“adherence”[Text Word]) AND (“malaria”[Text Word]) AND (“rapid diagnostic test”[Text Word])

The search yielded 189 records from which 57 duplicates were removed. After screening the records for relevant studies, 106 records were excluded. Of the remaining 26 records, 5 records were excluded because these did not provide data on non-RDT versus RDT settings (Abdelgader et al. BMC Public Health 2012, 12:11; Mangham et al. Trop Med Int Health 2012, 17(3): 330-42; Manirakiza et al. BMC Public Health 2012, 12:482; Rowe et al. Malar J 2009, 8:275; Ukwaja et al. Ethiop J Health Sci 2010, 20(3):179-83).

Results of the remaining 21 records are described here, showing studies performed in presumptive treatment (Table S1) and microscopy settings (Table S2), respectively.
