# Supplementary material for: Scale-up of Malaria Rapid Diagnostic Tests and Artemisinin-Based Combination Therapy: Challenges and Perspectives in Sub-Saharan Africa
Source: PLoS Med. 2014 Jan 21;11(1):e1001590. doi: 10.1371/journal.pmed.1001590 (PMC3897367; doi:10.1371/journal.pmed.1001590)
Supplement: Table S2 — Patients treated with antimalarials and antibiotics in studies comparing microscopy with RDTs. (DOC) [file pmed.1001590.s002.doc]

**Table S2.** Patients treated with antimalarials and antibiotics in studies comparing microscopy with RDTs

| **Study** | **Country** | **Study design** | **Treatment with antimalarials** | | | | **Treatment with antibiotics** | |
| --- | --- | --- | --- | --- | --- | --- | --- | --- |
|  |  |  | **Microscopy** | | **RDT** | | **Microscopy** | **RDT** |
|  |  |  | **% of slide positives (95%CI), n/N** | **% of slide-negatives** | **% of RDT-positives** | **% of RDT-negatives** | **% of slide-negatives** | **% of RDT-negatives** |
| 1 | Zambia | Cross-sectional, cluster sample survey | 100.0, 64/64 | 58.4 (47.7-69.4),  45/77 | 96.6 (93.6-99.5),  140/145 | 35.5 (28.6-42.5),  65/183 | NR | NR |
|  |  |  |  |  |  |  |  |  |
| 2 | Tanzania | Randomized trial | 98.3 (96.3-100.0), 171/174 | 50.8 (47.7-53.8),  523/1030 | 98.9 (97.5-100.0),  186/188 | 53.7 (50.6-56.8),  540/1005 | 51.0 (47.9-54.0),  525/1030 | 51.6 (48.6-54.7),  519/1005 |
|  |  |  |  |  |  |  |  |  |
| 3 | Kenya | Pre-post cluster randomized controlled trial | 59.2 (47.7-70.6), 42/71^a^ | 3.8 (1.0-6.6), 7/183^a^ | 56.7 (44.1-69.2), 34/60^b^ | 6.2 (2.9-9.4), 13/210^b^ | NR | NR |
|  |  |  |  |  |  |  |  |  |
| 4 | Ghana | Randomized controlled open label trial | 98.3 (97.2-99.3),  565/575 | 49.5 (46.8-52.2),  656/1325 | 98.2 (97.1-99.2),  587/598 | 46.0 (43.3-48.7),  597/1298 | 28.6 (26.2-31.0),  379/1325 | 28.8 (26.3-31.3),  374/1298 |
|  |  |  |  |  |  |  |  |  |
| 5 | Tanzania | Observational study | 100.0, 12/12 | 82.5 (77.0-88.0),  151/183 | 100.0, 26/26 | 16.6 (13.0-20.2),  67/404 | 62.8 (55.8-69.8),  115/183 | 92.8 (90.3-95.3),  375/404 |
|  |  |  |  |  |  |  |  |  |
| 6 | Tanzania | Cross-sectional surveys^c^ | 99, NR/370 | 53, NR/215 | 99, NR/126 | 7, NR/628 | 54, NR/215 | 78, NR/628 |
|  |  |  |  |  |  |  |  |  |
| 7 | Tanzania | Cross-sectional surveys | 60.0 (51.9-68.1), 84/140^d^ | 14.6 (10.2-19.1), 35/239^d^ | 71.8 (64.0-79.5), 94/131^e^ | 7.0 (4.2-9.9), 22/313^e^ | NR | NR |
|  |  |  |  |  |  |  |  |  |
| 8 | Zambia | Cross-sectional study | 66.1^f^ | NA | 26.6^f^ | NA | NR | NR |

RDT, malaria rapid diagnostic test; BS, blood slide; NR, not reported; NA, not applicable

^a^ Combined intervention and comparison arms, and blood slide and RDT-results pre-RDT and ^b^ post-RDT implementation

^c^ Data derived from before-and-after analysis based on repeated cross-sectional surveys

^d^ Combined blood slide and RDT-results pre-RDT implementation and ^e^ post-RDT implementation

^f^ Antimalarial drug treatments per facility-month

**Reference list**

1. Hamer DH, Ndhlovu M, Zurovac D, Fox M, Yeboah-Antwi K, et al. (2007) Improved diagnostic testing and malaria treatment practices in Zambia. JAMA 297: 2227-2231.

2. Reyburn H, Mbakilwa H, Mwangi R, Mwerinde O, Olomi R, et al. (2007) Rapid diagnostic tests compared with malaria microscopy for guiding outpatient treatment of febrile illness in Tanzania: randomised trial. BMJ 334: 403.

3. Skarbinski J, Ouma PO, Causer LM, Kariuki SK, Barnwell JW, et al. (2009) Effect of malaria rapid diagnostic tests on the management of uncomplicated malaria with artemether-lumefantrine in Kenya: a cluster randomized trial. Am J Trop Med Hyg 80: 919-926.

4. Ansah EK, Narh-Bana S, Epokor M, Akanpigbiam S, Quartey AA, et al. (2010) Rapid testing for malaria in settings where microscopy is available and peripheral clinics where only presumptive treatment is available: a randomised controlled trial in Ghana. BMJ 340: c930.

5. Bastiaens GJ, Schaftenaar E, Ndaro A, Keuter M, Bousema T, et al. (2011) Malaria diagnostic testing and treatment practices in three different Plasmodium falciparum transmission settings in Tanzania: before and after a government policy change. Malar J 10: 76.

6. D'Acremont V, Kahama-Maro J, Swai N, Mtasiwa D, Genton B, et al. (2011) Reduction of anti-malarial consumption after rapid diagnostic tests implementation in Dar es Salaam: a before-after and cluster randomized controlled study. Malar J 10: 107.

7. Masanja IM, Selemani M, Amuri B, Kajungu D, Khatib R, et al. (2012) Increased use of malaria rapid diagnostic tests improves targeting of anti-malarial treatment in rural Tanzania: implications for nationwide rollout of malaria rapid diagnostic tests. Malar J 11: 221.

8. Yukich JO, Bennett A, Albertini A, Incardona S, Moonga H, et al. (2012) Reductions in artemisinin-based combination therapy consumption after the nationwide scale up of routine malaria rapid diagnostic testing in Zambia. Am J Trop Med Hyg 87: 437-446.
